# In Situ Nitrogen Mineralization, Nitrification, and Ammonia Volatilization in Maize Field Fertilized with Urea in Huanghuaihai Region of Northern China

**DOI:** 10.1371/journal.pone.0115649

**Published:** 2015-01-30

**Authors:** Xuelin Zhang, Qun Wang, Jun Xu, Frank S. Gilliam, Nicolas Tremblay, Chaohai Li

**Affiliations:** 1 Incubation Base of National Key Laboratory for Physiological Ecology and Genetic Improvement of Food Crops in Henan Province, China, Collaborative Innovation center of Henan Grain Crops, Agronomy College, Henan Agricultural University, 95 Wenhua Road, Zhengzhou, 450002, China; 2 Department of Biological Sciences, Marshall University, Huntington, WV 25755–2510, United States of America; 3 Horticulture Research and Development Centre, Agriculture and Agri-Food Canada, Saint-Jean-sur-Richelieu, Qc, J3B3E6, Canada; Tennessee State University, UNITED STATES

## Abstract

Nitrogen (N) fertilization potentially affects soil N mineralization and leaching, and can enhance NH_3_ volatilization, thus impacting crop production. A fertilizer experiment with five levels of N addition (0, 79, 147, 215 and 375 kg N ha^-1^) was performed in 2009 and 2010 in a maize field in Huanghuaihai region, China, where > 300 kg N ha^-1^ has been routinely applied to soil during maize growth period of 120 days. Responses of net N mineralization, inorganic N flux (0–10cm), NH_3_ volatilization, and maize yield to N fertilization were measured. During the growth period, net N mineralization and nitrification varied seasonally, with higher rates occurring in August and coinciding with the R1 stage of maize growth. Soil NO_3_^−^-N contributed to more than 60% of inorganic N flux during maize growth. Cumulative NH_3_ volatilization increased with N additions, with total NH_3_ volatilization during maize growth accounting for about 4% of added N. Relative to the control, mean maize yield in the fertilizer treatments increased by 17% and 20% in 2009 and 2010, respectively. However, grain yield, aboveground biomass, and plant N accumulation did not increase with added N at levels > 215 kg N ha^-1^. These results suggest that the current N rate of 300 kg N ha^-1^ is not only excessive, but also reduces fertilizer efficacy and may contribute to environmental problems such as global warming and eutrophication of ground water and streams.

## Introduction

Nitrogen (N) fertilizer is used extensively to enhance crop production [1]. In China, applications of N fertilizers increased by 191% from 1981 to 2007, and reached 32.6 million tons per year, however, grain production increased by only 50% for the same period [2, 3]. In Huanghuaihai region, China, over 300 kg N ha^-1^ was routinely applied during the 120 days of maize growth, whereas the average yield of maize grains was only 5300 kg ha^-1^[4]. Increased N application not only reduces the economic efficiency of fertilizer, it may also exacerbate environmental problems, including eutrophication of aquatic ecosystems and increased emissions of greenhouse gases [2, 5].

Nitrogen mineralization is the key process for controlling bioavailability of N for plants. Soil N dynamics during plant growth are affected by many factors, including use of inorganic fertilizer [6, 7]. Many studies have examined the effects of N fertilizers on N mineralization, and have showed that inorganic N applications increase N mineralization [3, 8, 9]. Nitrification is a fundamental step in the N cycle, converting reduced N to oxidized N pools. Variation of nitrification with N fertilizer in agroecosystems is related to agronomic N-use efficiency (NUE) and environmental/human health problems [10, 11, 12]. Previous researchers have found that N fertilizer application stimulates the transformation of NH_4_^+^ to NO_3_^−^ (nitrification), increasing the likelihood of loss of inorganic N via leaching of NO_3_^−^ and/or gaseous N emissions (N_2_O), and reducing NUE in agricultural systems [10, 11]. Therefore, it is necessary to understand the effects of excess N fertilization on N mineralization and nitrification.

Ammonia volatilization from agricultural soils not only causes a direct loss of plant N, but it can also be a significant environmental concern for soil, air, and water quality [13, 14]. It is an important pathway of N loss in agroecosystems [15, 16], being as high as 50% in some systems [17, 18], and being positively correlated with rates of N application. Certainly, optimizing N inputs from fertilization while minimizing N outputs from NO_3_^-^ loss and NH_3_ volatilization are imperative for farming systems to be both economically and ecologically sound [19, 20].

Maize is a cost-effective supplementary feed for livestock, and generally responds sensitively to N fertilization. Although sufficient inorganic N supply for maize growth is crucial [3, 4], presently, applications of N fertilizer are typically excessive in the Huanghuaihai region of China, where high N rates return minimal increases in economic yield. Fertilizer N cost for maize was 27.1 kg of fertilizer N for every kg of plant N, far greater than the global average of 8 kg fertilizer N/kg plant N, which translates to 1 kg fertilizer N to produce 49 kg grain yield [21, 22, 23].

The purpose of this study was to quantify these relationships. We examined N mineralization, nitrification, and NH_3_ volatilization in maize field under urea fertilization in Huanghuaihai region of northern China. These results are intended to advance understanding of the impacts of inorganic fertilization on agricultural systems.

## Materials and Methods

Statement: The field experiment was carried out in Hebi Academy of Agricultural Sciences, Henan Province, China. The manager is Xiangwen Cheng, who issued field permits for sampling soils within this field site. There was no potential impact on any endangered or protected species among these sampling sites.

### Study site

This experiment was conducted during the maize growth season (From June to October) in 2009 and 2010 in Xun County (116°41′E, 41°02′N; 72.3 m above mean sea level), Henan Province, China. The soil is Eutric Cambisols with a sandy loam texture (FAO classification). Mean monthly temperature ranges from 21°C to 27°C and average monthly rainfall from 0.2 mm to 142 mm of rain from June to October. Generally, winter wheat is planted early October and harvested early June of next year in this region, and summer maize is sowed in middle June, and harvested in late September. Residues of both wheat and maize are removed after harvesting. Over 300 kg N ha^-1^ as urea is annually applied to the soil during maize growth period. Basic soil properties of the field were measured prior to the experiment. Average total N content using the Kjeldahl acid-digestion method was 0.12%, soil organic carbon by H_2_SO_4_–K_2_Cr_2_O_7_ oxidation from 1.4 to 2.7%, Alkaline-extractable N 47.8 to 70.9 mg kg^-1^, Olsen-extractable P 12.4–16.9mg kg^-1^, NH_4_OAC-extractable K 145–171 mg kg^-1^, and soil pH from 7.8–8.01.

### Experimental design

Maize cultivar (Xundan 20), the most popular hybrid in this region, was chosen and hand-planted at 67 500 plants ha^-1^ on June 12, 2009 and 2010, and harvested on September 29, 2009 and 2010.

A completely randomized block design was used in the experiment. Five N fertilizer (urea) treatments were established in 2009 and 2010: 0 kg N ha^-1^ (control—N0), 79 kg N ha^-1^ (N79), 147 kg N ha^-1^ (N147), 215 kg N ha^-1^ (N215), and 375 kg N ha^-1^ (N375). Four replicates were used with each treatment. Each plot was 3 m × 15 m with a minimum buffer zone of 0.5 m between plots. During maize growth period, N fertilizer was applied to 5 cm depth soil at V6 stage (collar of the sixth leaf visible, 23 days after sowing: [DAS23]) and R1 stage (silks: DAS55). On DAS23, 0, 45, 45, 45, and 45 kg N ha^-1^, and on DAS55, 0, 34, 102, 170 and 330 kg N ha^-1^ were applied to the N0, N79, N147, N215, and N375 plots, respectively. Phosphorus and potassium fertilizers were added at 40 kg ha^−1^ P_2_O_5_ as CaHPO_4_·2H_2_O and K_2_O kg ha^−1^ as KCl to each plot on DAS23. All fertilizers were applied to a 5 cm deep soil ditch between corn rows prepared by hand and then covered with soil.

### Net N mineralization, nitrification and N leaching

Net N mineralization rates were measured using the modified in situ soil core incubation method [24]. Two PVC cylinders, 4.3-cm diameter × 15-cm-deep, were placed into soil to a depth of 10 cm in each plot at each measurement period. One core (initial soil) was removed from each plot for determining initial concentrations of KCl-extractable NH_4_^+^-N and NO_3_^−^-N, whereas the other core (incubated soil) was left in the field for a measured amount of time. One resin bag (2.5 cm diameter × 0.2 cm thick) was placed on the top of this soil core to deionize deposition from air, and two other resin bags at the bottom of the core, with the upper one for capturing ions leached from the soil core and the lower one for deionizing nutrients from the below soil. Inorganic N (NH_4_^+^-N + NO_3_^−^-N) in the upper bag was used to calculate N flux from 0–10 cm surface soil layer. Each resin bag held approximately 2.5 g of sulfonic acid-based cation resin (HCR-W2, H^+^ form) and 2.5 g of a trimethylbenzyl ammonium-based anion resin (21 KCl form) (Dow Chemical, Calgary, Alberta, Canada).

Both initial and incubated soils were sieved (2 mm mesh) to remove large organic materials. Approximately 10 g of sieved fresh soil was extracted for measuring inorganic N contents with 50 mL of 2 M KCl for 1 h on a variable speed reciprocal shaker (Apparatus Co. Ltd. Changzhou, China). The resin bag was also extracted with 50 mL of 2 M KCl. The extracts from soil and resin bags were analyzed for NH_4—_N and NO_3—_N by a Segment Flow Analyzer (Scalar SAN^plus^, Netherlands). During maize growth period in 2009, the starting date of incubation was June 12 (DAS.1), July 15 (DAS. 34), August 1 (DAS.51), August 15 (DAS.65), and September 1 (DAS.82), and the ending date of incubation was July 14 (DAS. 33), July 31 (DAS. 50), August 14 (DAS. 64), August 31 (DAS. 81), and September 29 (DAS. 110), respectively. The incubation periods were June 12—July 14 (DAS.1–33), July 15- July 31 (DAS.34–50), August 1-August 14 (DAS. 51–64), August 15- August 31 (DAS.65–81), and September 1–29 (DAS.82–110), respectively. The incubation period for 2010 was about two weeks.

Net N mineralization was calculated as the difference between post- and pre-incubation inorganic N (NH_4_^+^-N + NO_3_^−^-N), while net N nitrification was calculated as the difference between NO_3_^−^-N concentrations. Net N mineralization and net nitrification rates (kg N ha^-1^ d^-1^) were respectively calculated by following equations:

$$\text{Net N mineralization}=\frac{P_{m1}+B_{m}-P_{m0}}{T}$$

$$\text{Net N nitrification}=\frac{P_{n1}+B_{n}-P_{n0}}{T}$$

where, P_m1_ and P_n1_ (kg ha^-1^) was soil inorganic N (NH_4_^+^+ NO_3_^−^) and NO_3_^−^-N after incubation on a dry-weight basis, and P_m0_ and P_n0_ initial values of soil inorganic N (NH_4_^+^ + NO_3_^−^) and NO_3_^−^-N, respectively. B_m_ and B_n_ were inorganic N (NH_4_^+^ + NO_3_^−^) and NO_3_^−^-N in the resin bag, and T was incubation period (d).

### NH_3_ volatilization

A semi open-static system was modified to measure NH_3_ volatilization [25]. An ammonia-trapping chamber was constructed with PVC pipe with 20 cm in diameter and 20 cm tall. The base of the chamber was inserted 5 cm into the soil. Sheet PVC tabs, welded to each of the inner walls of the chambers at two levels, supported two sponges for capturing volatilized NH_3_. The lower sponge was used to absorb NH_3_ volatilized from the soil surface in the cylinder and the upper sponge to absorb NH_3_ from outside the cylinder and to prevent its absorption by the lower sponge. The sponges were immersed with 20 mL glycerol phosphoric acid (5%, v/v, phosphoric acid and 4%, v/v, glycerol) prior to being put into the chamber. At DAS23 and DAS55, the device was installed after N fertilizer. The lower sponge was replaced with a new one after 1, 3, 7, 14, 21, and 28 d of N fertilizer. The sponges were then taken to the laboratory and extracted with 500 mL of 1 M KCl for 1 h. About 100 mL equilibrium liquids were sampled and stored in freezer before analysis using a TRAACS auto-analyzer.

### Maize sampling

Three uniform maize plants from each plot were harvested to determine aboveground biomass on DAS110 (R6 stage: physiological maturity) in 2009 and 2010, and the plants were divided into stem (stem and sheath), leaf, and ear. All samples were dried to stable weights at 70°C and the biomass was measured.

Grain yield was determined on DAS110 by using a 2-m ×7-m frame. After drying, the ear diameter, ear length, grain number per row, grain number per spike, cob diameter, cob mass, 100 kernel mass were determined for 10 plants per plot (data not shown).

### Statistics

A split—split plot analysis of variance was used to analyze their effects on soil net N mineralization, nitrification, soil inorganic N content, inorganic N flux (leaching), and NH_3_ volatilization rate. Whole plots in a randomized complete block design were used, with N rates as the whole plot factor, years as the subplot factor, and sampling dates as the sub-subplot factor. All statistical analyzes were performed with the General Linear Models. If the treatment main effect was significant, and interactions were non-significant, mean separation was carried out using the least significant difference (LSD) procedure on main effect means for treatments. If treatment and year interaction was significant, the LSD was applied to the treatment means separately for each year, with mean differences compared at *p* = 0.05. Pearson product-moment correlation was used to determine the relationship between maize yield, net N mineralization, and NH_3_ volatilization in each year. Non-linear regression was used to determine the relationship between maize yield, soil N mineralization, nitrification, NH_3_ volatilization, and nitrogen fertilization in 2009 and 2010. All statistical analyses were performed using SPSS 10.0 (Chicago IL, USA).

## Results

### Net N mineralization and nitrification and inorganic soil N content

**Fig 1:**
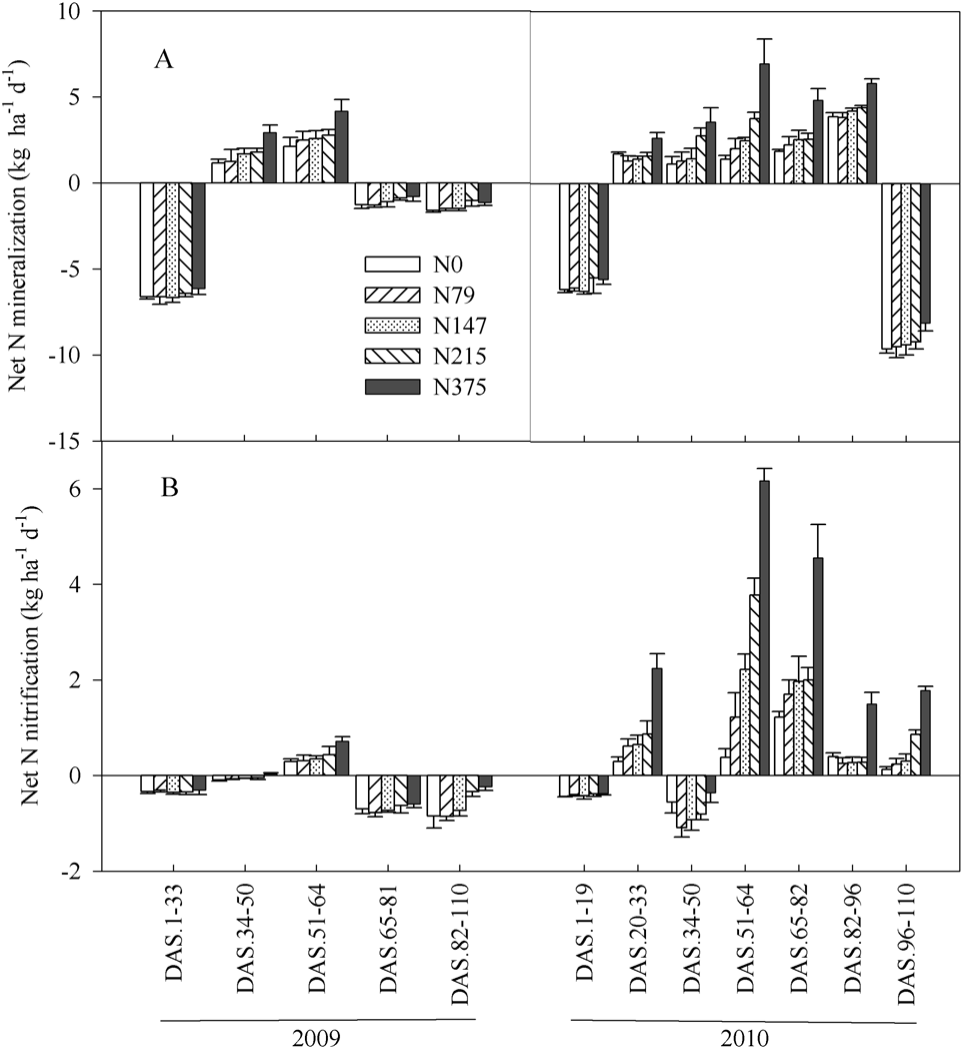
Soil net N mineralization (A) and nitrification (B) at different days after sowing (DAS) during maize growth in 2009 and 2010. Each point is the mean of 4 replicated plots. Error bars represent ± SE.

**Fig 2:**
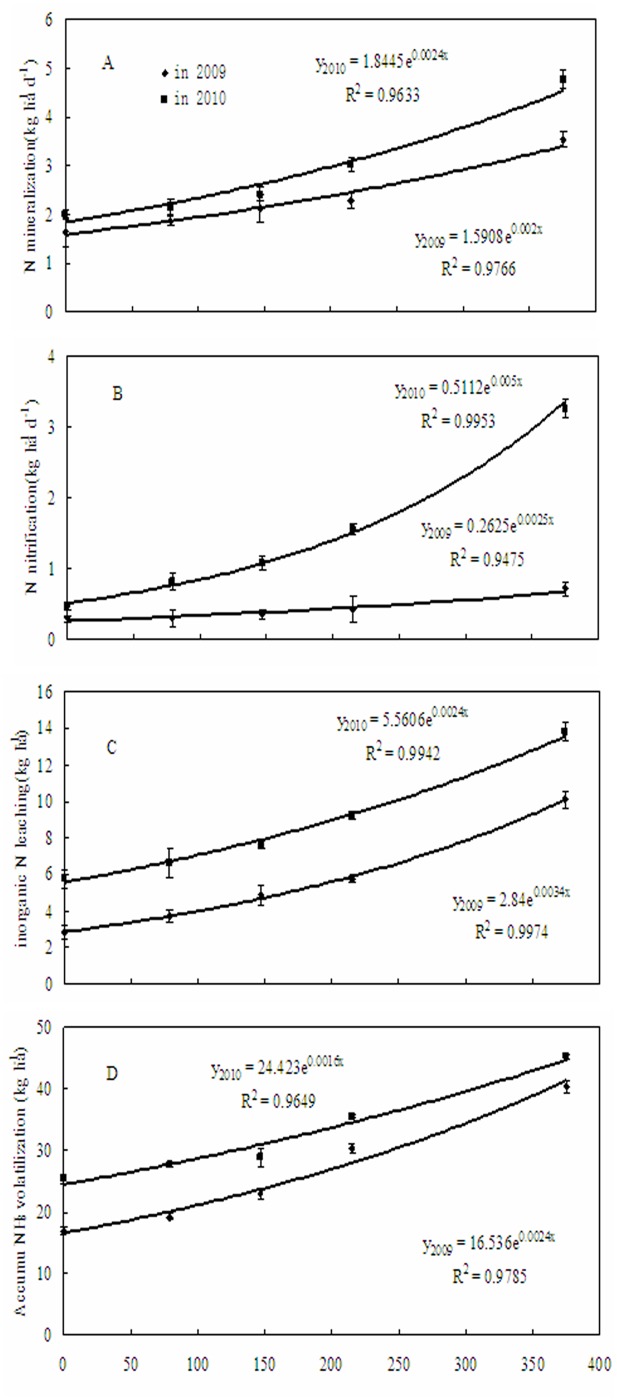
Cumulative net N mineralization, nitrification, inorganic N leaching and NH_3_ volatilization at different N rates during maize growth periods in 2009 and 2010. Each point is the mean of 4 replicated plots. Error bars represent ± SE.

Net N mineralization rate varied from-7.5 to 4.2 kg N ha^-1^ soil d^-1^ and from-11.1 to 10.6 kg N ha^-1^ soil d^-1^ during maize growth periods in 2009 and 2010, respectively. The difference of the rates was significant between 2009 and 2010 (Table 1 and Fig. 1). Greatest values occurred at DAS 51–64, corresponding to the maize silking stage (R1 stage) (Fig. 1A). Net N mineralization rates increased gradually with N rates at N rates less than 215 kg ha^-1^, while the differences between four N treatments were not significant. At 375 kg N ha^-1^, however, net N mineralization rates was 78% and 90% higher than the averages of other four treatments in 2009 and 2010, respectively. Net N mineralization was exponentially correlated with N rates (Fig. 2A), and significant for both 2009 and 2010.

**Table 1:** Results (*F* and *P* values) of the GLM ANOVA for the effects of N, years and sampling times on soil net N mineralization rate and nitrification rate, inorganic N content, inorganic N leaching, NH_3_ volatilization rate, corn yield, aboveground biomass and aboveground N accumulation.

Source	Net rate (kg ha^-1^ d^-1^)		Content (kg ha^-1^)		Leaching (kg ha^-1^)		NH_3_ volatilization (kg N ha^-1^ d^-1^)	Yield (kg ha^-1^)	Aboveground biomass (kg ha^-1^)	Aboveground N accumulation (kg N ha^-1^)
	mineralization	nitrification	NH_4_^+^-N	NO_3_^−^ -N	NH_4_^+^-N	NO_3_^−^ -N				
N	26.11***	59.33***	10.70***	4.89***	11.48***	66.04***	218.28***	10.65***	8.09***	5.99***
Yr	17.35***	427.25***	947.08***	29.32***	24.84***	3.03	150.91***	3.37	0.1	13.6***
ST	803.22** *	125.83***	383.32***	17.73***	19.69***	33.47***	159.56***			
Block	1.62	1.4	0.87	0.45	1.29	1.12	0.34	0.58	0.18	0.09
Yr * ST	253.52***	153.95***	417.62***	145.36***	9.38***	35.49***	33.25***			
N * Yr	5.43***	37.95***	1.02	8.69* **	2.22	2.08	2.12	0.09	0.08	0.59
N * ST	2.13**	9.33***	1.49	2.91* **	1.06	1.76*	9.13***			
N* Yr * ST	1.05	10.71***	1.22	3.48***	0.86	3.59***	2.36***			

N: Nitrogen fertilizer, Yr: Years, ST: Sampling times.

*, **, and *** indicated the significance at *p* = 0.05, 0.01 and 0.001 levels, respectively.

Net N nitrification rates were low in 2009, ranging from-1.59 to 0.93 kg N ha^-1^ soil d^-1^ (Fig. 1B), whereas in 2010, rates varied from 1.48 to 6.58 kg N ha^-1^ soil d^-1^. Soil net N nitrification increased exponentially with increasing N rates (Fig. 2B).

**Fig 3:**
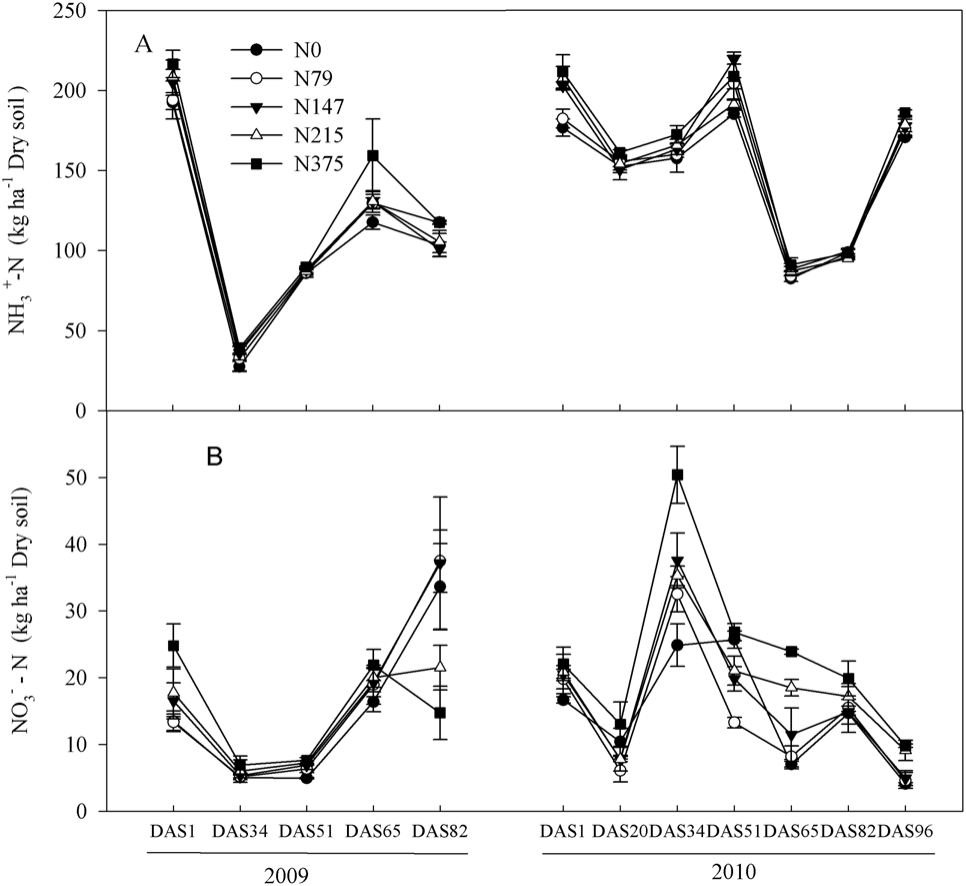
Dynamics of soil extractable NH4^+^-N (A), and NO3^-^-N (B) at different days after sowing (DAS) in the field trial during maize growth season (June.12—Sep.29) in 2009 and 2010. Each point is mean of 4 replicated plots. Error bars represent ± 1 SE.

Over the maize growth periods in 2009 and 2010, soil NH_4_^+^ -N contents varied from 22 to 236 kg ha^-1^ and from 75 to 251 kg ha^-1^ (Fig. 3A), respectively. Applying N fertilizer did not significantly affect soil NH_4_^+^ -N content. Relative to the control, mean NH_4_^+^ -N contents of four N fertilizer rates in 2009 and 2010 increased by only 10% and 7%. Soil NO_3_^-^-N contents varied among the N rates, ranging from 3 to 66 kg ha^-1^ in 2009 and from 2 to 63 kg ha^-1^ in 2010 (Fig. 3B).

### Soil N flux and NH_3_ volatilization

**Fig 4:**
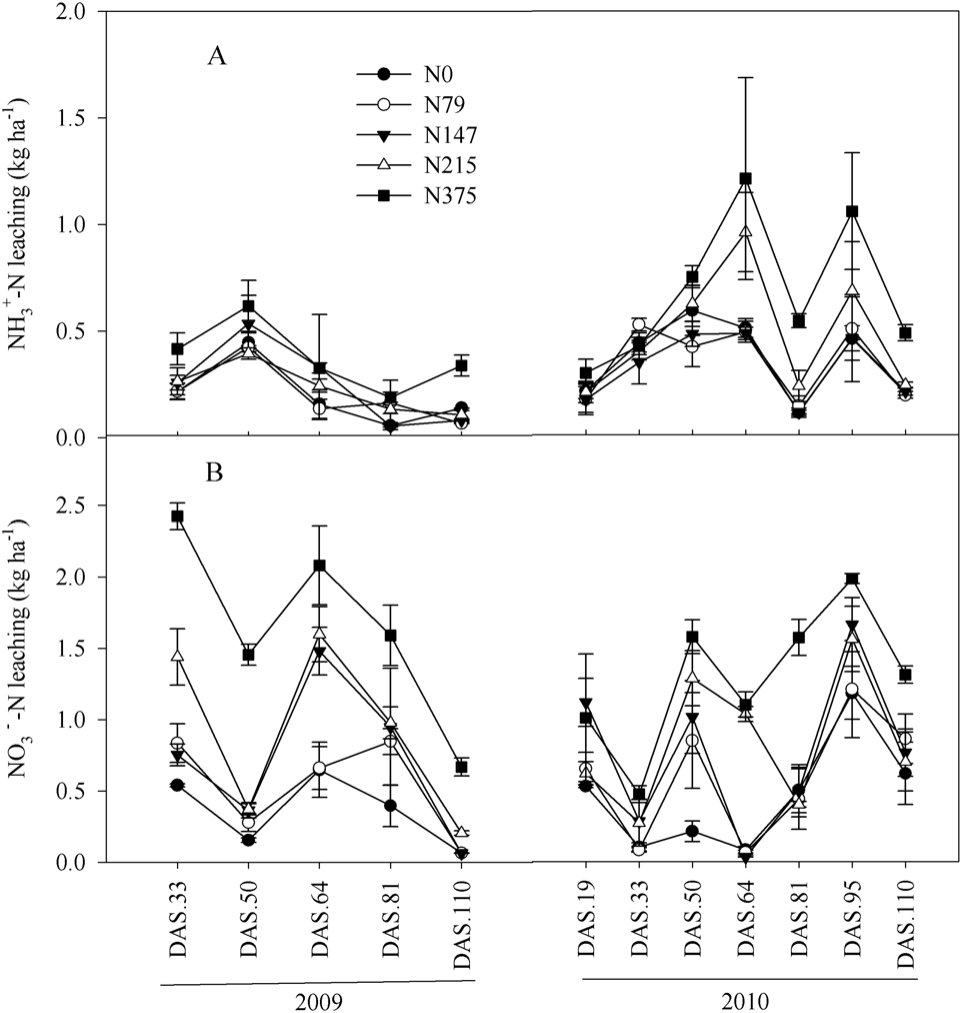
Leaching dynamics of NH4^+^-N (A) and NO3^-^-N (B) at different days after sowing (DAS) during maize growth in 2009 and 2010. Each point is mean of 4 replicated plots. Error bars represent ± 1 SE.

Soil NH_4_^+^_—_N and NO_3_^-^-N fluxes did not show consistent seasonal patterns during maize growth period (Fig. 4). However, mean soil inorganic N flux in the fertilizer treatments increased by 117% and 62% in 2009 and in 2010 in comparison with the control, respectively. Cumulative inorganic N flux exhibited an exponential relationship with N fertilizer rates (Fig. 2C). The correlation between soil inorganic N leaching and N mineralization was significant in both 2009 (*r* = 0.87**, n = 20) and 2010 (*r* = 0.92**, n = 20).

**Fig 5:**
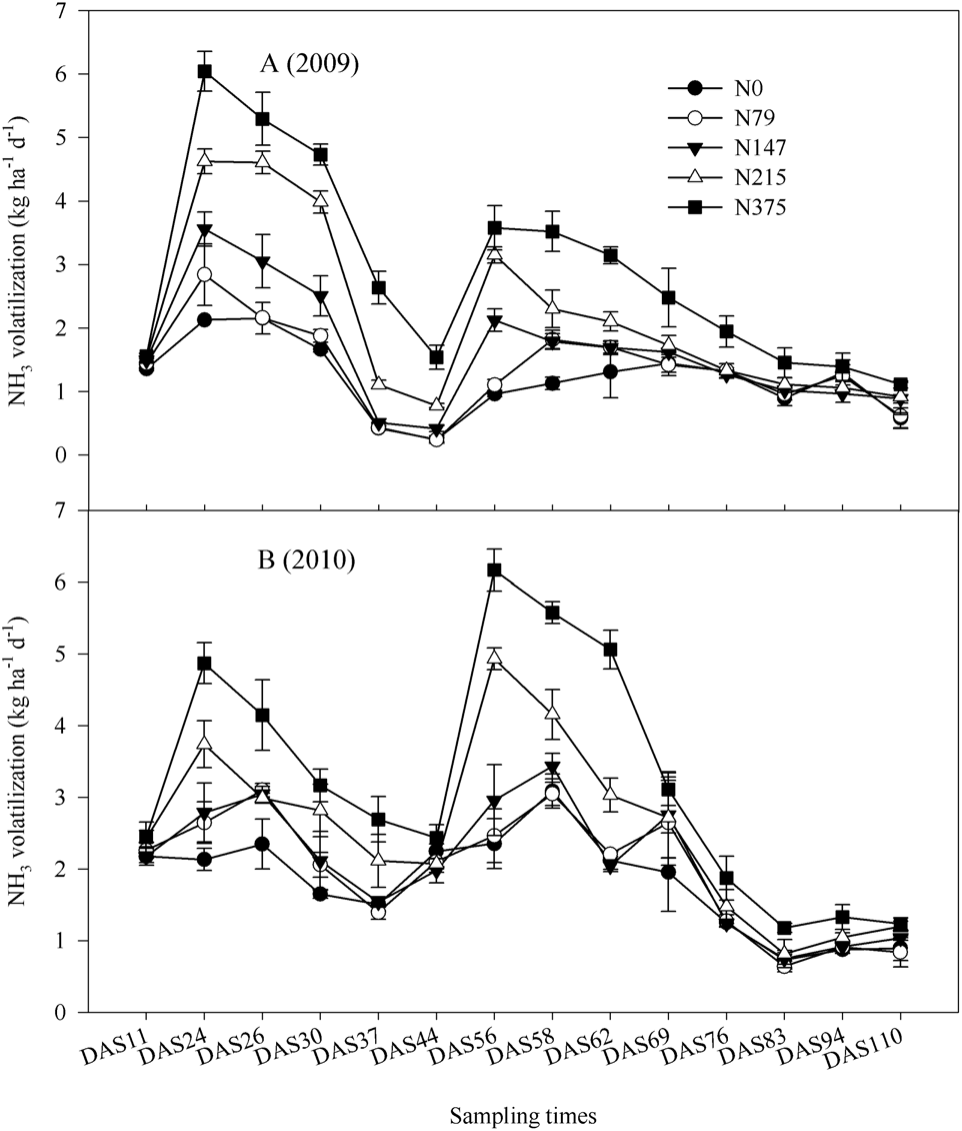
Ammonia volatilization (kg N ha^–1^ d^–1^) in five N fertilizer treatments at different days after sowing (DAS) during maize growth periods (June.12—Sep.29) in 2009 and 2010. The N fertilizer was incorporated to the soil at V6 stage (Days after sowing 23: DAS23) and R1 stage (Days after sowing 55: DAS55). Each point is the mean of 4 replicated plots. Error bars represent ± SE.

Soil NH_3_ volatilization rates under N fertilization treatments showed similar temporal variation in both 2009 and 2010 (Fig. 5A and B) during maize growth periods. The rates of NH_3_ volatilization varied significantly from 0.15 to 6.52 kg NH_3_ ha^-1^ d^-1^ in 2009 and from 0.23 to 6.98 kg NH_3_ ha^-1^ d^-1^ in 2010, and the higher rates occurred 1–7 days after N fertilization. Accumulated NH_3_ volatilization increased significantly with N fertilizer in both 2009 (*F*_4,19_ = 163.1, *P* = 0.001) and 2010 (*F*_4,19_ = 105, *P* = 0.001) (Fig. 2D). Mean accumulation of NH_3_ in 2010 was significantly higher than in 2009 (Table 1), with mean loss N via NH_3_ volatilization of four N fertilizer treatments accounted for 4% of the N fertilizer applied in 2009 and 2010. Soil NH_3_ volatilization rates were also significantly correlated with soil net N mineralization (*r*_2009_ = 0.81**, *r*_2010_ = 0.94**, n = 20) and nitrification (*r*_2009_ = 0.62**, *r*_2010_ = 0.96**, n = 20) in 2009 and 2010.

### Maize yield and N accumulation

**Fig 6:**
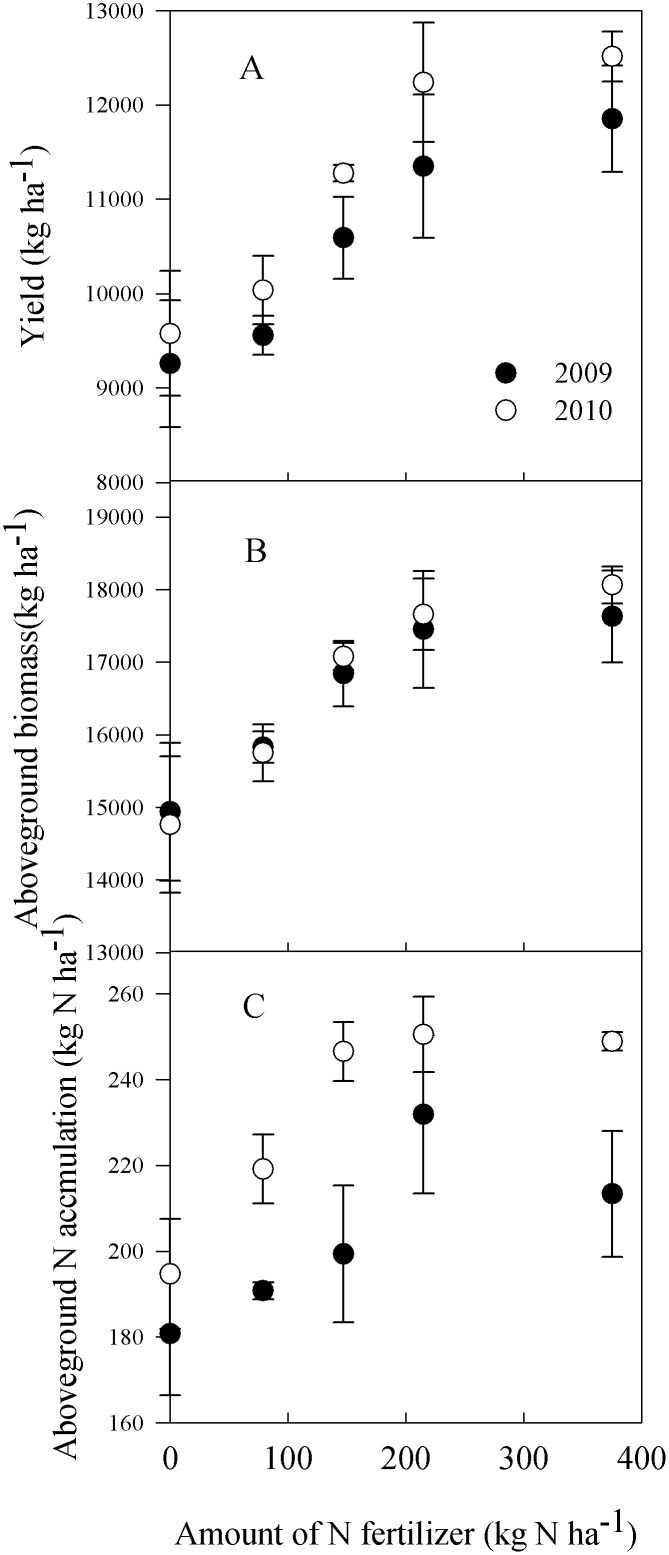
Maize grain yield, aboveground biomass and aboveground N accumulation of maize in five N treatments in 2009 and 2010. Each point is the mean of 4 replicated plots. Error bars represent ± SE.

Applications of N fertilizer significantly increased maize yields both in 2009 (*F*_4,19_ = 3.96, *P* = 0.02) and in 2010 (*F*_4,19_ = 8.08, *P* = 0.001). Mean yields of four N treatments in 2009 and 2010 increased by 17% and 20%, respectively, relative to the control. The model for maize yield and N addition over two years showed similar patterns, and neither grain yield nor aboveground biomass increased continuously when N addition exceeded 215 kg N ha^-1^ (Fig. 6A). Aboveground N accumulation showed similar trends to the yields. The mean N accumulation was 203 and 232 kg N ha^-1^ across the five treatments in 2009 and 2010, respectively. Likewise, the increments in plant N accumulation became small at N rates greater than 215 kg ha^-1^ (Fig. 6 C).

## Discussion

### Enhanced N mineralization and N flux under inorganic N fertilization

Our data show that N fertilization increased N mineralization, nitrification, inorganic N flux, and NH_3_ volatilization. Applications of an inorganic form of N can influence soil N transformations directly or indirectly through the alteration of biotic characteristics of soil and soil organic matter quality [26]. Direct effects include enhancing soil nutrition, increasing labile organic N, and stimulating microbial activity [27, 28]. Indirect effects arise primarily from variation of plant biomass among the fertilizer treatments. Because NO_3_^-^-N accounted more than 60% of inorganic N flux in mineral soil during maize growth (Fig. 4), this became a major pathway for N loss during this period [6, 11, 29]. Zhu et al. (2005) found that ~19% of N fertilizer applied to cropping systems in China was lost both to the atmosphere and water bodies [30]. Not only does high N leaching impact groundwater quality, it also represents a threat to human health [1, 31, 32]. Significant relationships between N mineralization and nitrification with N flux suggest that increasing N fertilizer application could increase N leaching [9, 33, 34]. Therefore, reducing nitrification and thus nitrate leaching could increase N utilization efficiency and reduce environmental risks.

Temporal variation of soil NH_3_ volatilization following N fertilizer application was consistent with previous studies that found highest NH_3_ volatilization rates occurring between a few hours to 12 d after N fertilization [13, 35, 36]. Such variation in agricultural soil is not only related to climatic factors and cropping system [36, 37, 38], but also to temporal patterns of soil N mineralization [39, 40]. Release of NH_4_^+^ from organic N stores usually predominates in the period after maximum volatilization of NH_3_ [41].

Both NH_3_ volatilization and soil NO_3_^-^ leaching may also have substantial relevance to regional biogeochemistry and global climate change [7, 13]. Ammonia is a chemically reactive gas, readily combining with NO_3_^-^ and SO_4_^2-^ in acid cloud droplets to form acidic aerosols, with subsequent deposition of these aerosols contributing to acidification and eutrophication of natural ecosystems [7]. Nitrous oxide (N_2_O) formed during nitrification, and from denitrification where there is excess NO_3_^-^, is an important greenhouse gas accounting for approximately 5% of the total greenhouse effect. Emissions of N_2_O likely enhance China’s overall contribution to anthropogenic global warming [16, 23].

### Reduced N economical efficiency under elevated fertilization

Data in this study demonstrate that maize yield and N accumulation did not increase proportionally with levels of N addition, both generally leveling off at 215 kg N ka^-1^. Accordingly, it is clear that with high levels of N addition, N utilization efficiency was reduced. Many factors may affect the utilization efficiency of fertilizer N, such as plant N uptake, photosynthetic potential, and grain N concentration [42, 43]. However, reduction of maize yield also indicated that the capacity for N uptake was limited when the N fertilizer was higher than the threshold. Nitrogen fertilizers are expensive inputs, costing agriculture more than US $45 billion per year [22]. Whereas more than 50% of the N applied to field is not assimilated by plants, the predicted yield production was not reached with such high N fertilizers inputs. The decline of cereal production per unit of applied N indicates higher economic and environmental costs for each unit of food produced [22, 44].

In conclusion, this study found that excessive N fertilization can greatly increase net N mineralization, NH_3_ volatilization, and inorganic N leaching, while reducing the economic efficiency of added N. Because applications of 215 kg N ha^–1^ did not increase maize yield and N uptake, rather increased N leaching and NH_3_ volatilization, from both agricultural and environmental perspectives, N fertilizer rate should be no more than 215 kg N ha^–1^.
